# Data handling strategies for high throughput pyrosequencers

**DOI:** 10.1186/1471-2105-8-S1-S22

**Published:** 2007-03-08

**Authors:** Gabriele A Trombetti, Raoul JP Bonnal, Ermanno Rizzi, Gianluca De Bellis, Luciano Milanesi

**Affiliations:** 1 Institute for Biomedical Technologies – National Research Council (ITB-CNR), via Fratelli Cervi 93, 20090 Segrate (MI), Italy; 2 Consorzio Interuniversitario Lombardo per l'Elaborazione Automatica (CILEA), via Raffaello Sanzio 4, 20090 Segrate (MI), Italy

## Abstract

**Background:**

New high throughput pyrosequencers such as the 454 Life Sciences GS 20 are capable of massively parallelizing DNA sequencing providing an unprecedented rate of output data as well as potentially reducing costs. However, these new pyrosequencers bear a different error profile and provide shorter reads than those of a more traditional Sanger sequencer. These facts pose new challenges regarding how the data are handled and analyzed, in addition, the steep increase in the sequencers throughput calls for much computation power at a low cost.

**Results:**

To address these challenges, we created an automated multi-step computation pipeline integrated with a database storage system. This allowed us to store, handle, index and search (1) the output data from the GS20 sequencer (2) analysis projects, possibly multiple on every dataset (3) final results of analysis computations (4) intermediate results of computations (these allow hand-made comparisons and hence further searches by the biologists). Repeatability of computations was also a requirement. In order to access the needed computation power, we ported the pipeline to the European Grid: a large community of clusters, load balanced as a whole. In order to better achieve this Grid port we created Vnas: an innovative Grid job submission, virtual sandbox manager and job callback framework.

After some runs of the pipeline aimed at tuning the parameters and thresholds for optimal results, we successfully analyzed 273 sequenced amplicons from a cancerous human sample and correctly found punctual mutations confirmed by either Sanger resequencing or NCBI dbSNP. The sequencing was performed with our 454 Life Sciences GS 20 pyrosequencer.

**Conclusion:**

We handled the steep increase in throughput from the new pyrosequencer by building an automated computation pipeline associated with database storage, and by leveraging the computing power of the European Grid. The Grid platform offers a very cost effective choice for uneven workloads, typical in many scientific research fields, provided its peculiarities can be accepted (these are discussed). The mentioned infrastructure was used to analyze human amplicons for mutations. More analyses will be performed in the future.

## Background

In 1986, around the time the Human Genome Project was initiated, the cost of sequencing was around $10 per base. By 2001, the cost had fallen to about 10 to 20 cents per nucleotide [1]. Nowadays, Sanger sequencing can be approached at a cost of around 0.5 cents per nucleotide (that's a 2000-fold drop) but a recent technology breakthrough, pyrosequencing, is likely to drop the costs even further, while simultaneously increasing the throughput by an order of magnitude or more. Pyrosequencing [2-5] is a real-time, sequencing by synthesis method based on the detection of released pyrophosphate during DNA synthesis. Pyrosequencing's most impressive feature is the throughput, being up to 10 megabases/hour. On the other hand, the sequenced fragments have reduced lengths compared to Sanger ones, being 94 bases on average in our experience.

The recent dramatic increase in sequencing throughput together with the reduction of costs calls for increased computation power, as well as increased storage space, in order to keep up. Also, it is to be considered that most bioinformatics tasks such as genome assembly, inversion distance computation, genome rearrangement analysis and molecular dynamics have got a quadratic or higher complexity.

In addition, an analysis of the CPU speed trends reveals that the CPU speed increases are considerably lower nowdays than they used to be in the past. For example let's consider AMD CPU release history:

- June 2000: Athlon Thunderbird 600 released [6] (a 600+ CPU by definition of AMD PR rating which is based on the Athlon Thunderbird performances)

- February 2003: Athlon XP Barton 2500+ released [7] (a 2500+ CPU)

- May 2005: Athlon 64 3800+ released [8] (a 3800+ CPU)

Between June 2000 and February 2003 (32 months) there was a speedup of 4.2× making an average speedup of 71% per year, while between February 2003 and May 2005 (27 months) the speedup was a mere 1.5× making an average speedup of 21% per year. Some hope for new performance improvements is brought by recently marketed multi-core CPUs, however at this point in time it is still not clear how quickly these can evolve (e.g. how quickly the number of cores can increase). Note that we quoted AMD's CPU history and not Intel's just because AMD names its CPUs against a Performance Rating (PR), which is a better indicator of effective CPU speed than clock speed that Intel uses, and hence makes it easier to compare CPU performances through time and across architectural improvements.

Keeping up with bioinformatics data is hence becoming increasingly difficult in the localized environment. A computing cluster might seem the solution, however for small companies and small research groups producing uneven spikes of computationally intensive jobs, a privately owned cluster might not be an effective solution as it tends to be either very expensive or underpowered during the actual spikes of work, while remaining underused for the majority of the time.

In the aforementioned situation, the European Data Grid (EDG [9]), a large community of computation clusters – load balanced as a whole, is likely to offer a better alternative. After formally requesting access to the Grid to INFN [10], certificates will be issued and the new grid user can leverage the power of more than a thousand CPUs spread all over Europe. The Grid power is not completely free: after a significant submission of jobs, INFN might ask the new user to share some computing resources, however, the overall hardware cost would still be very low compared to that of a dedicated cluster able to handle a significant workload.

Grid job submission itself is rather simple, however the strict limitation in the size of the input sandbox (1 MB for data and executables) and other subtleties described hereafter in this paper can discourage a regular use of the Grid.

Our first contribution to this paper is the development of a computation pipeline, integrated with a database system, for storing and analyzing human amplicons sequences coming from a high throughput 454 Life Sciences GS 20 [4] pyrosequencer.

The pipeline started as localized and was then ported to the Grid. To ease the porting to the Grid we developed Vnas, a Grid job submission, virtual sandbox manager and job callback framework, which constitutes our second contribution to this paper. Vnas is aimed at rendering the Grid job submission significantly more powerful yet simpler, and allows to overcome the Grid submission limitations without affecting the Grid infrastructure negatively.

## Results and discussion

### Amplicons experiment

Initially, we extensively leveraged the "repeatability with altered parameters" feature together with the "by hand searchability" of the results database mentioned in the Methods section. This allowed us to easily compare results obtained with various parameter sets, fine-tune the parameters and thresholds for our pipeline and better understand the peculiar behaviour of our new 454 Life Sciences GS 20 pyrosequencer (property of CNR ITB).

Afterwards, we successfully analyzed 273 sequenced amplicons from chromosomes 1, 3, 5, 9, 11, 13, 17, 18, 19 of a cancerous human sample and correctly found punctual mutations confirmed by either NCBI dbSNP or Sanger resequencing. More analyses are expected for the future.

The Grid porting for this pipeline is discussed in the Vnas sections.

### Vnas framework and pipeline Grid porting

We report below the average times for the first computation step (the most CPU intensive).

#### 1- First computation step for a dataset using 20 slices: time gain factor = 5

The computation step 1 input was divided into 20 slices, each slice was submitted to the grid then results were fetched back. The whole process was repeated multiple times to calculate the following averages:

##### Submission time

16 seconds per slice (Grid job). This involves fetching of sequence data, creation of sandbox implying some copying/linking of directory trees and files (custom), gzip compressions and md5 hash computations (by Vnas), actual submission of the job to the Grid (by Vnas).

##### Queue time

1 minute to 2+ hours, 5 minutes on average for one job, 12 minutes is the average for the maximum wait time on a set of 20 jobs. For a discussion on the great variance of the queue time in the EDG, see the related paragraph in Appendix B.

##### Execution time

7 minutes per slice (but slices are executed in parallel).

##### Total time (average case)

20 × 16 sec + 12 min + 7 min ~= 24 minutes

The total time for the first computation step is 24 minutes on average instead of 120 minutes seen in local execution. Similarly happens for the other steps for which we omit timing measurements. This is already a significant improvement, and more interestingly, our local computation resources remain free during most of the time.

#### 2- First computation step for 10 datasets using 10 × 4 slices: time gain factor = 15

The grid is more effective when more data can be processed in parallel. This is the case when we have to recompute a number of old datasets with altered pipeline parameters. The following are average time measurements for a recomputation of 10 datasets like the above one. In this case we used fewer, bigger slices (4 slices per dataset – 35 minutes of execution time for one slice) so as not to flood the Grid with too many jobs and also to reduce the maximum queue wait time.

##### Submission time

22 seconds per slice (job)

##### Queue time

the average for the maximum wait time on a set of 40 jobs is roughly 30 minutes

##### Execution time

35 minutes per slice (but slices are executed in parallel)

##### Total time (average case)

40 × 22 sec + 30 min + 35 min = 80 minutes

The total time here is 80 minutes, instead of 20 hours seen in local execution. This is clearly a great improvement compared to local execution. One could submit even more computation data: the greater the computation you can submit which can execute in parallel, the higher the benefit you get from the Grid.

In Appendix B you can find some more details regarding Grid queues times. In Appendix C you can find some observations on the difficulties which still persist when porting applications to the Grid.

## Conclusion

We have shown how we realized a multistep computation pipeline integrated with a database system to analyze punctual mutations from human amplicons, taking into consideration the peculiarities of the new pyrosequencer, and how we ported such pipeline to the European Data Grid to leverage its enormous computing power obtaining a speedup factor up to 15. We have also shown how the Grid can be economically very advantageous for the small and medium research group producing uneven spikes of workload compared to a dedicated cluster.

We have shown how the porting of a parallelized application to the Grid can be significantly eased with our newly developed Vnas framework, providing a virtual sandbox of unlimited size with support for complex directory structures, multiuser transparent file sharing over the Grid with timed expiration, and a callback mechanism for a higher automation of join points in computation pipelines. Vnas is currently in beta stage but will soon be available for download. We expect the ease of use of Vnas to help increase the usage of the European Grid with profit also to the bioinformatics community.

## Methods

### Methods for Amplicons project

This project consisted in the creation of a computation system for the analysis of human amplicons sequenced by a 454 Life Sciences GS 20 high throughput pyrosequencer for finding punctual mutations, as well as a storage system for both GS 20 sequence data and computation results. More specifically, our requirements were the following:

- Support for heterozygosity in punctual mutations (this is not supported by some other approaches or softwares such as the software bundled with the 454 Life Sciences GS 20 pyrosequencer)

- Coping with the peculiarities of the 454 GS 20 pyrosequencer, mainly:

- Shorter reads than with a Sanger sequencer: (94 bases on average)

- Different error profile, in particular, problems basecalling near homopolymers

- Large amount of data: throughput up to 10 MBases/hour

- Demonstrability: ensure repeatability of every computation allowing demonstration of every result obtained

- Altered parameters: allow repeatability of every computation with altered parameters, constants and thresholds in order to be able to compare the results

- Searchability: possibility to perform flexible hand-made searches on every sequence data (sequences and their quality), every old and recent final computation result and every intermediate meaningful computation result

- Storage requirements: the storage system needed was required to store at least the following entities:

- Multiple projects of analysis on the same data

- Biological samples and protocols

- Sequences read by the GS20 sequencer for each run

- Multiple co-existing databases of reference sequences

- Final and intermediate computation results

- Incremental backups: need to backup the storage system incrementally

The analysis system was also required to be applicable to a much larger kinase analysis project in the future.

**Figure 1:**
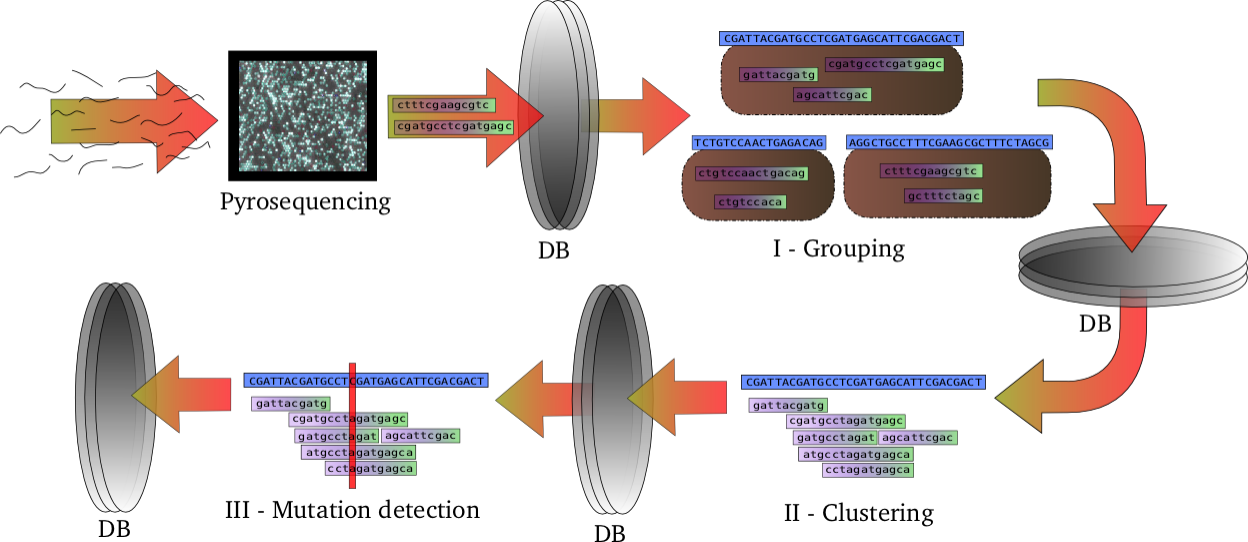
**Amplicons computation pipeline**. Step I groups sequenced amplicons according to their most similar reference sequence (or sequences: more than one reference accepted in the case of close similarity of references). Step II clusters groups of sequences belonging to the same references while applying certain thresholds and heuristics. Step III detects punctual mutations and avoids false positives due to homopolymers. A database stores sequence data and computation results (intermediate and final), and fully separates the steps of the pipeline.

Due to the large amount of expected data and the above requirements such as the "Altered Parameters" which foresaw the need to repeat huge amounts of past computations within a reasonable time, even though our first implementation was localized we immediately planned the computation system for future execution on the Grid environment. Hence we implemented a 3-step computation pipeline implying two join points (between steps I and II and between steps II and III). At the beginning and at each join point data are fetched from the storage database and fed to the computation stage. At the end of each computation stage the results are stored back into the database. There is no direct communication between the stages, this was done in order to ensure complete separation between the stages allowing a simpler migration to the Grid. The pipeline is illustrated in fig 1.

The database was implemented in MySQL, featuring 11 tables. The results are retrievable via SQL queries to permit advanced hand-made and currently unforeseeable researches by the biologists. The repeatability with altered parameters is allowed by the tables structure which allows the association of multiple computation projects to the same sequenced data. The consistency is ensured by the transactional and referential integrity capabilities of MySQL. The incremental backups are performed using rdiff-backup.

Let's now quickly outline how we addressed the above mentioned peculiarities of the 454 pyrosequencer:

• Different error profile: a heuristic algorithm implemented in step 3 ensures that nearby homopolymers do not trigger false punctual mutations. The algorithm is very reliable, however, it is currently based on the assumption that there are no indels in the genomic material hence it does not currently support indels.

• Short reads: The higher speed and lower operation costs of the GS 20 machine allowed us to use a high coverage, hence discriminating sporadic errors due to random noise from heterozygous mutations in most cases. The required multiplicity is around 10 × for a 95% certainty. In the case that similar regions are present on more than one reference sequence, a heuristic algorithm in step 2 discriminates which matches are to be kept and which ones are to be discarded.

This pipeline was later ported to the Grid. This is described in the Vnas sections.

### Methods for the Vnas framework

Notwithstanding the advantages of the Grid mentioned in the Background section, some issues exist that can discourage a regular use of the Grid.

Problems related to grid jobs submission are the following:

• Limited sandbox: Users of the Grid have got a limited space to store data and executables they need for their job. Current limit is 1 MB for most users. All files not fitting into 1 MB need to be uploaded on Storage Elements (SEs) separately by hand, and need to be downloaded on the worker node prior to execution. Files stored on SEs need to be deleted by hand when no longer needed or they would waste shared resources.

• Flat sandbox and Storage Elements: The Grid sandbox and the SEs can only bear raw files. If a job needs a nested directory structure this has to be created with a custom code on the job. Tar archives are also to be packed and unpacked manually.

• Needless reuploading of the same files: If many users need the same files, every user will need to upload the files on SEs unbeknownst to the others, wasting bandwidth and storage space.

• Slices management: Commonly, in order to improve performances on the Grid, conceptually monolithic jobs are first divided into computation steps by creating a pipeline, and then each pipeline step is split in smaller "slices" which can execute in parallel. Slices are then submitted to the Grid separately. Unfortunately, the code to poll and wait for all slices to complete execution, fetch results, and launch the next computation stage is to be created as custom code for each application, which is considerably time consuming for the developer.

In order to overcome the aforementioned problems, during the grid porting of our Amplicons pipeline we developed Vnas: a framework for Grid job management providing the following innovative features:

• **Virtual Sandbox: **sandbox emulation providing a sandbox of unlimited(*) size also allowing nested subdirectories. This prevents (1) the need to manually upload big files to storage elements (2) the need to pack and upload archives for recreating directory structures needed for the job to run and (3) the need to write code in the job for downloading the files described at points -1- and -2- and for unpacking the archives at point -2- prior to starting the computation.  (*) Please note that while there is no limitation imposed by Vnas on the size of the Virtual Sandbox, the Virtual Sandbox is only an abstraction substituting a manual upload of the files onto the Storage Elements. The Virtual Sandbox does not create any new storage space on the Grid and the user is still responsible for the files uploaded by Vnas in this way. The user who has uploaded a file to the Grid is always traceable by the Grid administrators.

• **Job completion callback: **this allows a custom command to be executed by Vnas when a certain set of slices completes execution (Vnas constantly monitors the submitted jobs). This can be used to trigger the following step in a computation pipeline and releases the pipeline programmer from needing to write such code.

• **Automatic deletion of Grid uploaded files **that have not been used recently. This prevents the waste of Grid storage resources due to users forgetting to remove files they no longer need.

• **Grid bandwidth optimization: **the Vnas-uploaded files are left on the Grid Storage Elements for a certain time frame before deletion: this prevents a needless reupload of the same files in case these are needed by the same or even by another Grid user within the time frame. This prevents a waste of Grid bandwidth.

• **Sharing of files uploaded by different Grid users: **files uploaded by Vnas for Virtual Sandboxes are never uploaded by Vnas more than once: if a second job submission, even by a different Grid user, needs a file that has already been uploaded file its presence is detected on the Grid and the file is not uploaded again. This saves users' time and Grid storage space. The files uploaded to the Grid by Vnas are identified by their md5 hash. This allows Vnas to identify files with certainty based on their content and regardless of the name the users locally assigns to the files (which could be different for the same file for different Grid users). The md5 hash also prevents false positive matches that can arise due to random name equality among files or due to a file existing in different versions with the same name.

In Appendix A you can find details about the functioning of Vnas.

After the development of Vnas, we leveraged Vnas to port our Amplicons analysis pipeline onto the Grid in a significantly shorter time than would otherwise be needed. The virtual sandbox feature was particularly useful for us, in fact the Amplicons pipeline consists of various executables which alone would result in a 7 MB upload in addition to Biopython libraries requiring a deeply nested structure, and a significant amount of sequence data and computation results for each slice. The job completion callback was also needed, and if we hadn't implemented it separately in Vnas, we would have had to implement it anyway at every step of the pipeline. A discussion on some of the results obtained is located in the Results section.

## List of abbreviations and uncommon terms used

**EDG **- European Data Grid

**Grid **- Network of computing clusters and storage elements, load balanced as a whole. For the purpose of this paper the EDG is implied

**CE **- Computing Element: a cluster belonging to the Grid

**SE **- Storage Element: a storage resource of the Grid, usually located near a CE

**UI **- User Interface: computer acting as a grid client, mainly used for submitting jobs

**Vnas **- Vnas's Not A Submit: the new Grid job submission, virtual sandbox manager and job callback framework being presented in this paper

**Slice **- for the purpose of this paper: when a large job is subdivided and submitted as N separate Grid jobs, these are called slices. Most often the subdivision is made by splitting its input in N equal parts.

## Authors' contributions

GAT worked on the bioinformatics part of the amplicons project and implemented the grid Vnas framework. RJPB worked on the bioinformatics part of the amplicons project and also interfaced with the sequencing/chemical team. ER worked at the sequencing/chemical part of the amplicons project providing sequence data. GDB coordinated the sequencing/chemical part and gave guidelines for the bioinformatics part of the Amplicons project. LM coordinated the bioinformatics part of the amplicons project and the development of the Vnas grid framework.

## Appendix A – Vnas functioning

**Figure 2:**
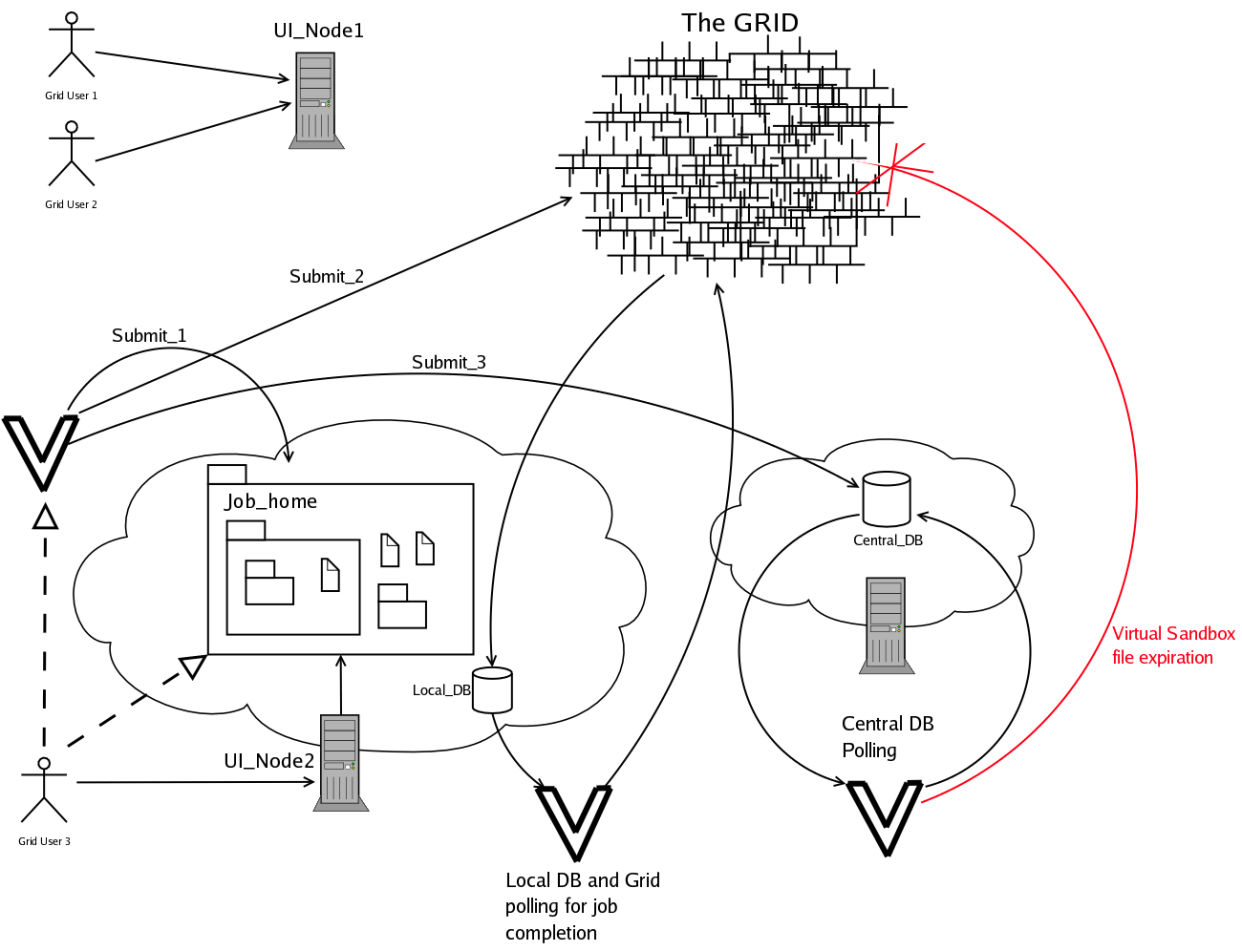
**Vnas distributed architechture**. The figure shows, from right to left: A) A Vnas loop on the central database, detecting expired virtual sandbox files and removing them from the Grid. B) A Vnas loop on the local UI database and the Grid, polling for the status of the most recently submitted jobs. C) A job submission on UI2 by GridUser3, and more in detail: C1) User creates a job_home. C2) User invokes Vnas. C3) Vnas scans job_home (Submit_1), uploads the files to the grid (Submit_2) and creates the corresponding entries or updates the ''last usage'' timestamps for the uploaded files, on the Central Database (Submit_3).

Vnas architecture (fig 2) is decentralized, with the exception of a central database containing hashes of files submitted for Vnas virtual sandbox functionality. The central database should (but is not required to) be shared among a high number of Grid users to better take advantage of the sharing of files uploaded by different users. The Vnas architecture ensures that the per-user load on the central database is very low hence the system can still scale linearly up to a large number of users (in the thousands).

Vnas job submission is instead performed from standard Grid User Interface (UI) nodes. A local database located on the same host stores Vnas information about job submissions and users' callback requests.

Vnas has the following three main operating modes:

- Job submit *(direct user invocation)*

- Job run *(triggered by the grid infrastructure)*

- Set-callback request *(direct user invocation)*

### Outline of job submit

• The user creates a sample job_home directory containing all the files needed for the job to run, i.e. both executables and data, then s/he invokes Vnas.

• Vnas scans the job_home directory, packs files and subdirectories of job_home separating them in sub-archives and single files according to a customizable algorithm.

• Vnas then computes the md5 hashes of all such files and archived contents, and uploads them onto the grid with a filename generated from their hash (some might exist already: the upload is skipped). Then Vnas contacts the central database for updating the "last access" timestamp for the uploaded files. A small set of files up to a certain (user definable) amount of KB are packed into a physical (traditional) sandbox.

• Vnas then submits the job to the grid, and records the job information such as the grid job identifier on the local database.

### Outline of job run

• A "jobprepare" script (belonging to the Vnas distribution, and automatically inserted into the job submission by Vnas) is run first. The jobprepare scans some bundled data to find the configuration of the job_home to be recreated.

• Jobprepare downloads all the needed archives and single-files to recreate the content of the job_home directory tree. The downloading is performed in a write-through fashion: if the file's nearest replica is still geographically distant, it gets replicated from there onto the Storage Element closest to the worker node, then gets downloaded on the worker node. The latter happens on a local network, and is basically immediate. This kind of replication ensures that subsequent downloads of the same files are faster and faster, and that the more a file is used, the higher the number of replicas. Space does not risk to get wasted for long, as when a file remains unused for a number of days, all replicas get deleted by a Vnas instance polling the central database.

• Vnas invokes the user specified executable and waits for its termination

• Vnas packs the user-requested result files and uploads them onto a storage element, using a filename automatically generated at the time of job submission.

### Outline of set-callback request

• The user invokes Vnas specifying a set of job identifiers that s/he wishes to wait for, and the command to be invoked by Vnas when the condition arises. Vnas records such information into the local database.

### Vnas polling loops

In addition to the three above described main working modes, two additional slow paced Vnas polling loops are needed in order to provide the virtual sandbox file expiration and the callback functionalities:

• A very slow paced Vnas loop on the central database node, which monitors the "last access" timestamps of files uploaded by UI Vnas instances, for virtual sandbox functionality. Vnas clears the Grid from all replicas of files which were last used too long ago. The exact number of days for expiration can be configured but values between between 7 and 15 days are typical. Longer times save Grid bandwidth at the expense of storage space and vice versa.

• A medium paced Vnas loop on each UI interface, which polls the local database and the Grid information system to fetch the state of the last jobs submitted by the users of the node. When all the jobs required for a certain callback request complete their execution, the user command is invoked with the credentials of the user who requested it (leveraging sudo -b). This is typically used to trigger a further step in a computation pipeline.

## Appendix B – EDG queue times

In general, the longer the job can be executed without join points (involving the collection of results of all the previous slices and resubmission of the new step), the more advantageous the Grid submission, since the queue time has to be waited for a smaller number of times.

Another observation is that, intuitively, the average queue time (intended as the average of the worst time on N slices) and the execution time should be balanced in an optimal job submission: the higher the number of slices, the lower the computation time but the higher the chance that one of them ends up in an unfortunate queue hence raising the global wait time. Raising the number of slices too much will end up raising the total perceived execution time for your job as a whole instead of lowering it.

The reason for the great variance in the queue time is partly in the random load experienced by the EDG and partly due to the "push" working mode of the EDG. Push mode means that a job is assigned to a CE (Computing Element) at the time of job submission. Even though a heuristic algorithm estimates the response time of the various CE before assigning the job, the heuristic is based solely on the queue length at each CE calculated in terms of number of jobs. Since there is no way for a queued job to declare its estimated running time, there is risk for an incoming job to be assigned to a queue containing few very long jobs, which would make its queue time unfairly long.

The EDG staff is working to implement a "pull" algorithm in which jobs join a centralized queue waiting to be pulled by the first free CE. This would effectively level unfairly long waiting times. The due time for the pull algorithm is not known to us. In the meanwhile, a newly implemented Vnas feature allows the automatic issuing of the job cancel and requeueing when the waiting time exceeds a user specified threshold. By the time the reissuing is made, the previous CE will have lengthened its queue, hence the job gets requeued on a different CE. In the future Vnas might resubmit the job without issuing a job cancel to the previous job, then closely monitor the two jobs and cancel one of the two as soon as the other enters execution.

## Appendix C – Persisting obstacles in porting applications to the Grid

One of the obstacles found when porting an application to the Grid which still persists and that could deter a more widespread usage of the Grid is the burdensome splitting of input data to create multiple slices of a conceptually single job (even though for the implementation of the "wait all results" join the Vnas callback can be leveraged), and the consequential parsing of the results from multiple slices at the end, for recreating a single result set. Unfortunately, it is not easy to conceive a tool which can help with this in the general case, since the way to perform splitting of data and merging back of results depends heavily on the type of data and the specific problem. Of course, a similar problem would also arise in a privately owned cluster.
